# Two Giant Cystic Uterine Adenomyomas in a Premenopausal Woman: The Largest Case to Date With Immunohistochemical Findings

**DOI:** 10.1155/crog/4595994

**Published:** 2026-01-21

**Authors:** Kazuhisa Kitami, Toshihide Matsumoto, Seigi Furukawa, Toshio Takada, Itaru Sanoyama, Makoto Saegusa, Yoshiki Murakumo, Kazuyoshi Kato

**Affiliations:** ^1^ Department of Obstetrics and Gynecology, Kitasato University School of Medicine, Sagamihara, Kanagawa, Japan, kitasato-u.ac.jp; ^2^ Department of Pathology, Kitasato University School of Medicine, Sagamihara, Kanagawa, Japan, kitasato-u.ac.jp

**Keywords:** adenomyosis, cystic adenomyoma, endometriosis, uterine adenomyoma, uterus

## Abstract

**Background and Aims:**

Cystic adenomyoma is a rare focal cystic variant of adenomyosis, and giant lesions are particularly uncommon. Malignant transformation has been reported in endometriosis‐related disease, but the molecular features of cystic adenomyoma, especially in adults, remain unclear. We are aimed at describing a premenopausal patient with two giant cystic adenomyomas, including the largest lesion reported to date, and to explore a possible pathogenic mechanism using immunohistochemistry.

**Methods:**

A 47‐year‐old nulliparous premenopausal woman presented with progressive abdominal distension and urinary symptoms. Imaging showed two large hemorrhagic cystic masses adjacent to a mildly enlarged fibroid uterus, and ovarian endometriotic cysts were suspected preoperatively. The patient underwent hysterectomy with bilateral salpingo–oophorectomy. Gross, histologic, and immunohistochemical examinations were performed on the uterus and cystic lesions. Clinical follow‐up was obtained for 10 months.

**Results:**

Surgery revealed two cystic adenomyomas measuring 30 and 10 cm, contiguous with the uterus but separate from both ovaries. The thick cyst walls were composed of smooth muscle bundles, and the inner surfaces were lined by a single layer of endometrial‐type epithelium; multiple foci of conventional adenomyosis were also present. In the cystic adenomyomas, glands were HNF‐1*β*+, pAKT+, estrogen receptor+, PTEN−, PIK3CA−, and ARID1A− with a p53 wild‐type pattern. Eutopic endometrial glands were HNF‐1*β*+, PIK3CA+, pAKT+, ARID1A+, and p53+, with mixed PTEN‐positive and ‐negative glands. The postoperative course was uneventful, and no recurrence was observed at 10 months.

**Conclusion:**

This case represents the largest cystic adenomyoma reported to date and the first adult case characterized in detail by immunohistochemistry. Differential PTEN and ARID1A expression between eutopic endometrium and cystic adenomyomas supports a model in which PTEN‐deficient endometrial clones invade the myometrium to form adenomyosis, with additional ARID1A loss and pAKT activation driving cystic enlargement without malignant transformation.

## 1. Introduction

Adenomyosis of the uterus is a common disorder prevalent in women in their 40s or those who are premenopausal and being treated for abnormal uterine bleeding or secondary dysmenorrhea. Adenomyosis is histologically characterized by the presence of endometrial glands and stroma within the myometrium [1]. Nodular adenomyosis consists of circumscribed nodular aggregates known as adenomyomas, which are rarely accompanied by cystic adenomyoma, which comprises a cystic cavity lined by normal endometrial cells filled with a hemorrhagic “chocolate‐like” fluid.

Focal cystic variants of adenomyosis have been described in the literature under various overlapping terms, including “cystic adenomyoma,” “cystic adenomyosis,” and “adenomyotic cyst”. In this report, we use the term “cystic adenomyoma” to refer to a cystic lesion within the myometrium measuring ≥1 cm in diameter, lined by endometrial‐type epithelium and filled with hemorrhagic content, corresponding to what has also been termed cystic adenomyosis or adenomyotic cyst in previous reports.

Cystic adenomyoma, which presents as a 1–2 cm cystic lesion within the myometrium of the uterus in young patients with a chief complaint of severe dysmenorrhea beginning at menarche, has been reported as juvenile cystic adenomyoma [2, 3]. Some researchers have hypothesized that juvenile cystic adenomyoma results from impaired development of the Müllerian ducts, leading to duplication or persistence of paramesonephric tissue; [4] others consider it a cystic variant of uterine adenomyosis [3]. Hormonal therapy with GnRH agonists and oral contraceptives is generally reported to be less effective in relieving symptoms [3], whereas surgical excision of the cystic adenomyoma by laparoscopy, robot‐assisted surgery, or hysteroscopy is effective in improving pain.

In contrast, the more rare cystic adenomyoma occurs in women in their 40s and is characterized by subserosal polypoid lesions larger than those found in the juvenile cystic adenomyoma that grow into the peritoneal cavity. Owing to their rarity, the pathogenesis of cystic adenomyoma remains poorly understood; with an etiology differing from that of the juvenile form, it is hypothesized to be caused by endometrial injury invagination [5]. Previous uterine surgery may be the pathologic basis for this disease [6]. As for treatment, total hysterectomy is often the procedure of choice, as fertility preservation is often not desired at the age of diagnosis.

In this study, we report a case of two giant cystic adenomyomas, 30 and 10 cm in size, the largest adenomyomas reported to date, and suggest a pathogenic mechanism as revealed by immunohistochemistry (IHC). Our aim is to improve the understanding of this rare condition. This study was conducted according to the principles of the Declaration of Helsinki. Written informed consent for publication was obtained from the patient.

## 2. Case Presentation

A 47‐year‐old nulliparous premenopausal woman was referred to our hospital with abdominal distention, frequent urination, and bilateral lower limb edema. She had first noticed the abdominal distention 1 day before presentation and visited a local gynecologic clinic, from which she was referred to our institution. She reported a history of only mild dysmenorrhea that did not require analgesics and denied any chronic pelvic pain. Pelvic ultrasonography revealed two cystic masses 30 and 10 cm in diameter filled with homogeneous low‐echogenic fluid content and adjacent to the uterus. Magnetic resonance imaging (MRI) revealed two well‐circumscribed cystic masses containing T1 high‐signal intensity and T2 low‐signal intensity (Figures 1a, 1b, 1c, and 1d). Laboratory examinations on admission revealed anemia. Serum carcinoembryonic antigen and carbohydrate antigen 19‐9 (CA19‐9) levels were within the normal limits; however, carbohydrate antigen 125 (CA 125) level was elevated to 60 U/mL. Although both cystic masses appeared contiguous with the uterine surface, a mildly enlarged uterus containing several small fibroids could be clearly delineated separately from the two cysts. In addition, the cysts contained blood‐like fluid, and their relatively thick but smooth walls did not show obvious continuity with the myometrium, making a myometrial origin less likely on imaging. Taken together, these preoperative findings favored a diagnosis of ovarian endometriotic cysts; therefore, a hysterectomy with bilateral salpingo–oophorectomy was performed.

Figure 1(a) Sagittal view of T2‐weighted MRI showing a well‐circumscribed cystic adenomyomas (arrows) containing low‐intensity area, contiguous with the uterine anterior and posterior wall. (b and c) Axial view of T2‐weighted MRI showing cystic adenomyomas (arrows), contiguous with the right wall of the uterus (arrowheads). (d) Axial view of T1‐weighted MRI showing cystic adenomyomas (arrow), contiguous with the right wall of the uterus (arrowheads).

**Figure:**
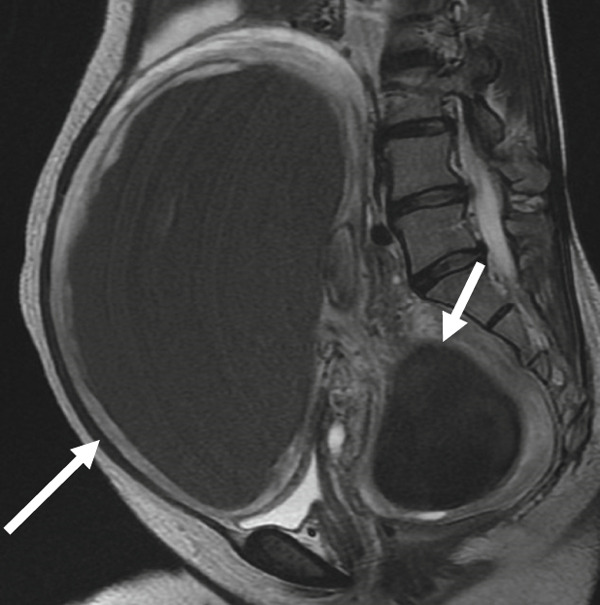
(a)

**Figure:**
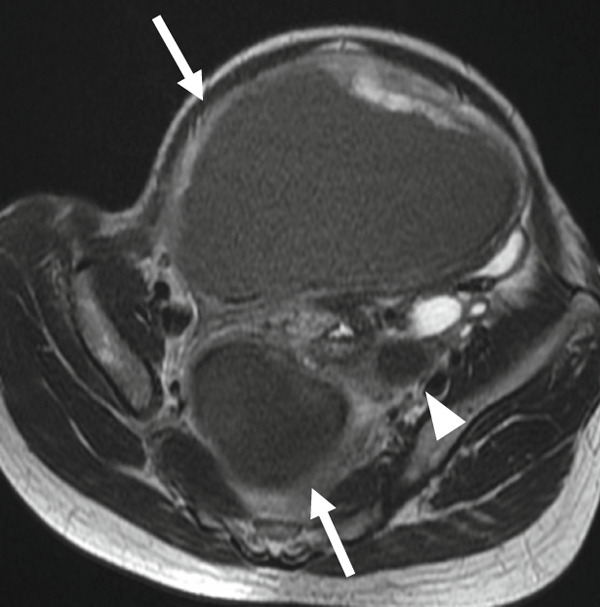
(b)

**Figure:**
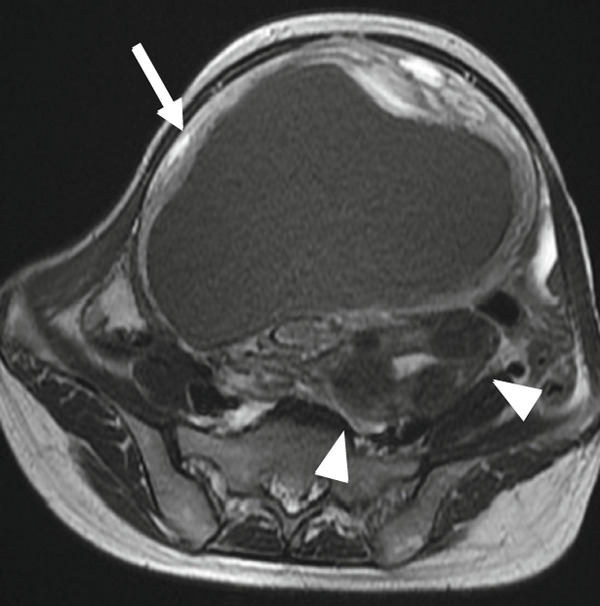
(c)

**Figure:**
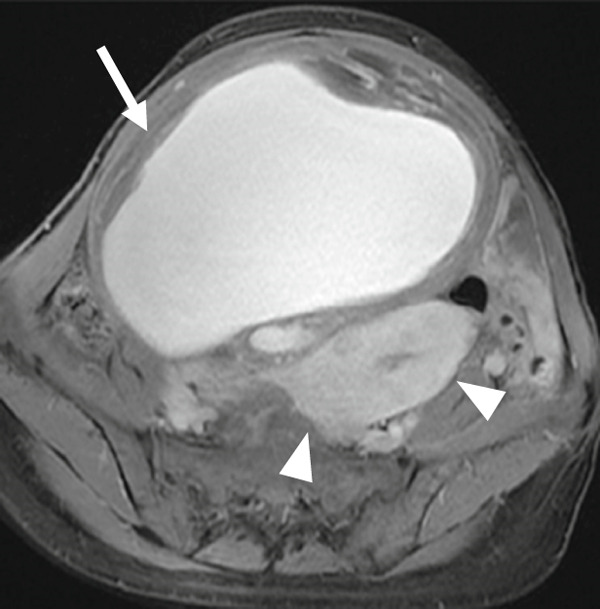
(d)

A cystic adenomyoma 30 cm in diameter was located on the anterior side of the uterus, and another cystic adenomyoma 10 cm in diameter was located in the Douglas pouch contiguous with the uterus. These masses were separated entirely from the bilateral ovaries (Figure 2a). The cystic adenomyoma of the Douglas pouch occupied the pelvic cavity and was challenging to remove; hence, the contents of the cystic adenomyoma were aspirated and excised along with the uterus. The contents comprised a viscous, brown, and bloody fluid.

Figure 2(a) Macroscopic appearance of the cystic adenomyomas, the excised uterus (arrowhead), and bilateral adnexa (short arrows). (b) Cross‐section of the excised uterus (arrowhead) and cystic adenomyomas (arrows). Scale bars = 5 cm. (c and d) Histologic findings of the resected cystic adenomyoma. (c) The inner surfaces of the larger cyst. (d) The inner surfaces of the smaller cyst. (e and f) The uterine endometrial epithelium. (Hematoxylin & eosin; scale bars = 50 *μ*m).

**Figure:**
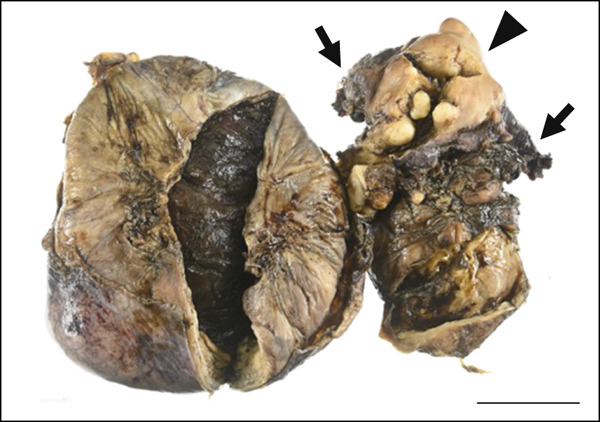
(a)

**Figure:**
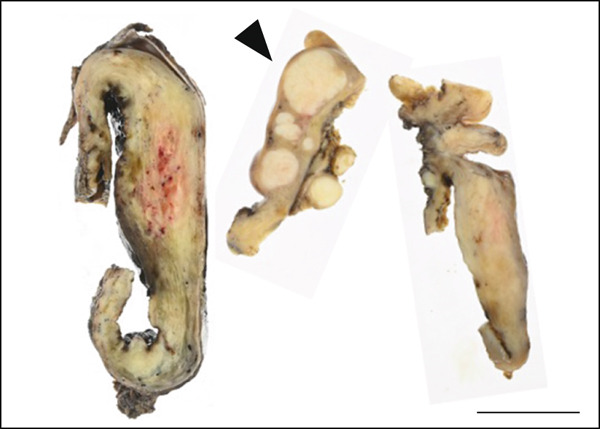
(b)

**Figure:**
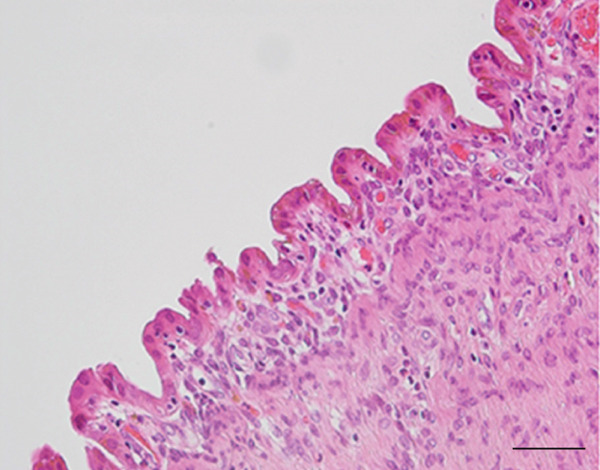
(c)

**Figure:**
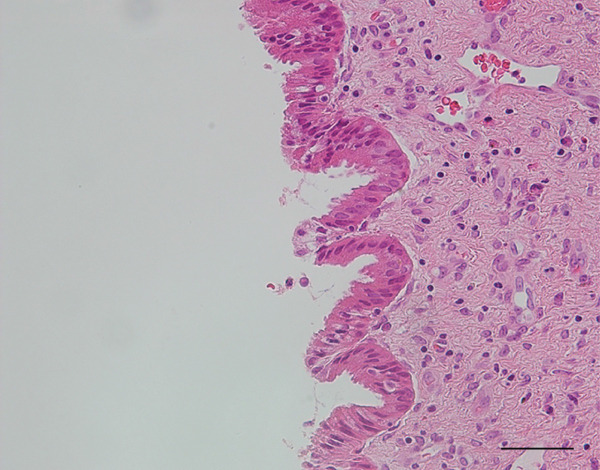
(d)

**Figure:**
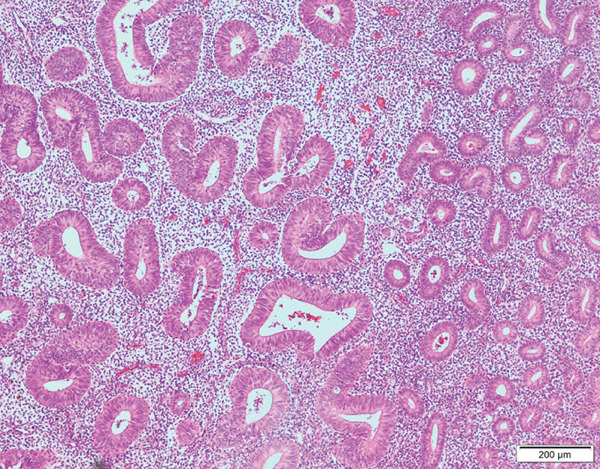
(e)

**Figure:**
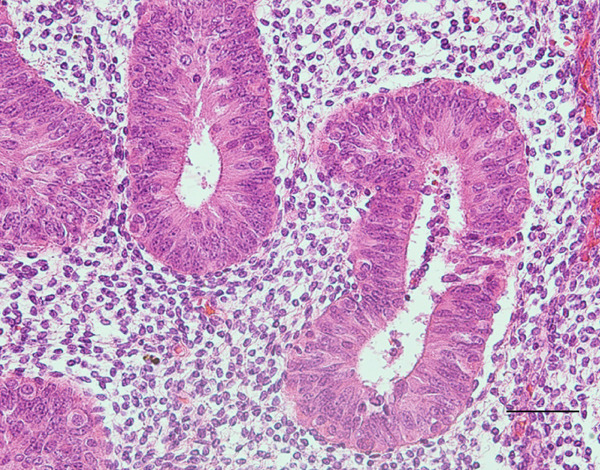
(f)

**Figure 3:**
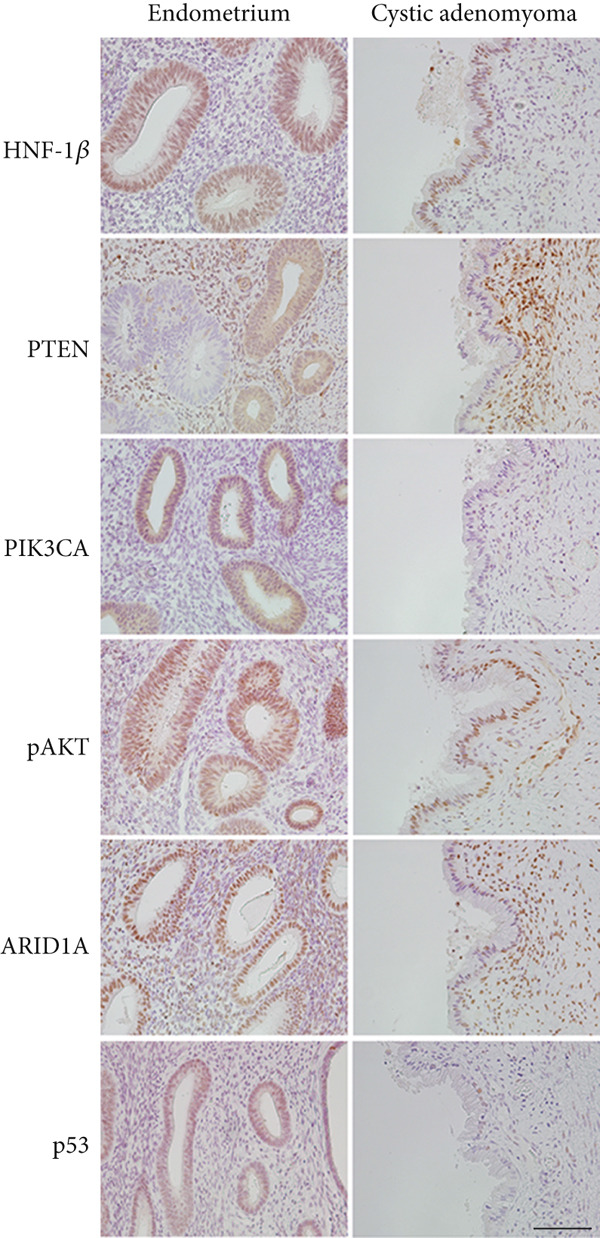
Immunohistochemistry for HNF‐1*β*, PTEN, PIK3CA, pAKT, ARID1A, and p53 in the endometrium and the epithelium within the cystic adenomyoma. Scale bar = 100 *μ*m.

Probing revealed no communication between the endometrial cavity and lumen of the two cystic adenomyomas (Figure 2b). Multiple uterine fibroids were observed in addition to the adenomyomas. Histologically, in addition to the cystic adenomyomas, multiple foci of conventional adenomyosis—characterized by endometrial glands and stroma within the myometrium—were observed in the excised uterine muscle layer, with 3–5 foci per ×40 field (Figures S1a, S1b, and S1c). The thick walls of the large cysts were composed of smooth muscle bundles. The inner surfaces of these cysts were lined with a single layer of glandular epithelial cells similar to those of the endometrial epithelium (Figures 2c, 2d, 2e and 2f). In the stroma of the epithelial cells, CD10‐positive stroma cells were detected. IHC revealed that the endometrial glands within the cystic adenomyomas were positive for hepatocyte nuclear factor‐1*β* (HNF‐1*β*), phospho‐AKT (pAKT), and estrogen receptor; negative for PTEN and phosphatidylinositol 3‐kinase catalytic subunit alpha (PIK3CA); and practically negative for AT‐rich interactive domain 1A (ARID1A). They also showed a p53 wild‐type staining pattern and PCR results showed no PIK3CA mutations. Bilateral ovaries and fallopian tubes showed unremarkable findings. In contrast, the uterine endometrial gland cells were positive for HNF‐1*β*, PIK3CA, pAKT, ARID1A, and p53. A mixture of PTEN‐positive and negative endometrial glands was observed (Figure 3).

The postoperative course was uneventful. The patient showed no signs of recurrence at 10 months postoperatively and the follow‐up was completed.

## 3. Discussion

The present case of two giant cystic adenomyomas merits discussion owing to the following factors: (i) the presence of two giant adenomyomas is extremely rare; (ii) this case contains the largest cystic adenomyoma reported to date; and (iii) no cases of cystic adenomyomas characterized by IHC have been previously reported

Cystic adenomyoma has not yet been clearly defined, and similar lesions have been reported under various names, including cystic adenomyosis and adenomyotic cysts. Endometrial cysts up to 2 cm in diameter within the uterine myometrium of juvenile patients are considered “juvenile cystic adenomyoma”; moreover, those that are not juvenile may also be considered cystic adenomyoma. Cystic adenomyomas that are not juvenile are relatively large. Previously, the largest reported cystic adenomyoma measured 20 cm in diameter; [7] in contrast, the largest lesion in our case measured 30 cm. In addition to those with thick muscular layers as described in this case study, cases of multiple cysts with thin subserosal capsules have also been reported [8].

Malignant tumors derived from endometriosis such as uterine carcinoma arising from adenomyosis and ovarian cancer (i.e., clear cell carcinoma or endometrioid carcinoma) arising from the ovarian endometrial cyst are common and have been previously reported. However, since cystic adenomyomas are rare, malignant transformation of cystic adenomyomas is also rare, with only three cases having been reported (two endometrial carcinomas and one clear cell carcinoma) [9–11]. Therefore, we performed IHC analysis in this case to determine whether the uterine cystic adenomyomas in this patient were benign but exhibiting tumor‐promoting changes similar to those of endometriosis‐derived malignant tumors. In our case, some uterine endometrial glands and the epithelium within the cystic adenomyomas revealed PTEN loss. Although ARID1A expression was positive in the uterine endometrial glands, it was lost in the endometrial glands of cystic adenomyomas. Recently, basic studies using tissue‐clearing techniques revealed that adenomyosis is induced by direct invasion of the endometrial glands and that endometrial gland cells within adenomyomas possess the same genetic mutations as the endometrial glands from which they invade [12]. In the present study, we did not perform IHC analysis on the adenomyotic areas identified within the myometrium. However, based on prior studies, it is reasonable to infer that these lesions may exhibit a pattern of immunohistochemical expression similar to that of eutopic endometrial glands, including partial loss of PTEN expression. On the other hand, a whole‐exome sequencing study of ovarian clear cell carcinoma has demonstrated clonal continuity among the uterine endometrium, endometriosis, and carcinoma, with increasing mutant allele frequencies of shared somatic mutations progressing from the endometrium, through distant and adjacent endometriosis, and ultimately to carcinoma [13]. By analogy, it is conceivable that a similar stepwise accumulation of somatic mutations—from eutopic endometrial glands to adenomyosis and subsequently to cystic adenomyomas—may underlie the pathogenesis and progression of the lesions observed in the present case. Further studies involving molecular analyses will be needed to verify this hypothesis. Furthermore, although PTEN knockout mice did not induce endometriosis carcinogenesis, endometriosis carcinogenesis was induced in 42% of PTEN and ARID1A double‐knockout mice [14]. Based on these and our IHC results, we speculated the occurrence of PTEN loss in some uterine endometrial glands, with the clones being the source of adenomyosis and partial ARID1A loss causing gigantism. Incomplete ARID1A loss may explain why it does not cause carcinogenesis. However, in an IHC study of endometrial carcinoma arising from uterine adenomyosis, ARID1A and PTEN expression was preserved ,whereas a p53 mutation was observed, and its histology was mostly endometrioid carcinoma grade 3 [1]. The tumor diameter was not large (4–5 cm) suggesting that p53 mutations in the early phase may have caused carcinogenesis. In this case, no p53 mutations were detected. Based on these reports, we speculated that our case was not oncogenic because of the absence of a p53 mutation, despite the loss of PTEN and partial loss of ARID1A which resulted in gigantism.

Ovarian endometrioid carcinoma and clear cell carcinoma vary regarding the molecular events that occur during endometriosis, which is the source of development; moreover, increased AKT phosphorylation was observed in endometriosis coexisting with ovarian clear cell carcinoma [15]. Our case, in which increased AKT phosphorylation was induced by loss of ARID1A rather than by PIK3CA mutation, could have resulted in clear cell carcinoma if not treated.

This report has several limitations. First, it describes a single case, and the proposed pathogenic mechanism based on immunohistochemical findings remains speculative and cannot be generalized to all cystic adenomyomas. Second, we did not perform comprehensive genomic analyses, such as next‐generation sequencing, and therefore cannot directly demonstrate clonal relationships or the stepwise accumulation of somatic mutations among eutopic endometrium, adenomyosis, and cystic adenomyoma in this patient. Further studies involving larger case series and integrated molecular profiling will be needed to validate and extend our observations.

In conclusion, this case represents the largest reported cystic adenomyoma to date. This is the first report describing the pathogenesis of giant cystic adenomyoma using IHC. We speculated that the cystic adenomyoma was enlarged by mutations in the proliferative signals of the endometrial gland cells. Future research should focus on elucidating the molecular biology of cystic adenomyoma, a task that is challenging owing to its rarity.

## Consent

Written informed consent was obtained from the subject.

## Disclosure

All authors have read and approved the final version of the manuscript. Kazuhisa Kitami had full access to all of the data in this study and takes complete responsibility for the integrity of the data and the accuracy of the data analysis. Kazuhisa Kitami affirms that this manuscript is an honest, accurate, and transparent account of the study being reported; that no important aspects of the study have been omitted; and that any discrepancies from the study as planned (and, if relevant, registered) have been explained.

## Conflicts of Interest

The authors declare no conflicts of interest.

## Funding

No funding was received for this manuscript.

## Supporting information


**Supporting Information** Additional supporting information can be found online in the Supporting Information section. Figure S1: (a–c) Histological findings of adenomyosis. Hematoxylin & eosin staining. Scale bars: (a) 1 mm; (b) 200 *μ*m; (c) 100 *μ*m.

## Data Availability

All data supporting the findings of this study are included in this article and its supporting information; no additional datasets are available.
