# Supplementary material for: Two Giant Cystic Uterine Adenomyomas in a Premenopausal Woman: The Largest Case to Date With Immunohistochemical Findings
Source: Case Rep Obstet Gynecol. 2026 Jan 21;2026:4595994. doi: 10.1155/crog/4595994 (PMC12820572; doi:10.1155/crog/4595994)
Supplement: Supplementary file 1 — Supporting Information Additional supporting information can be found online in the Supporting Information section. Figure S1: (a–c) Histological findings of adenomyosis. Hematoxylin & eosin staining. Scale bars: (a) 1 mm; (b) 200 μm; (c) 100 μm. [file CROG-2026-4595994-s001.pdf]

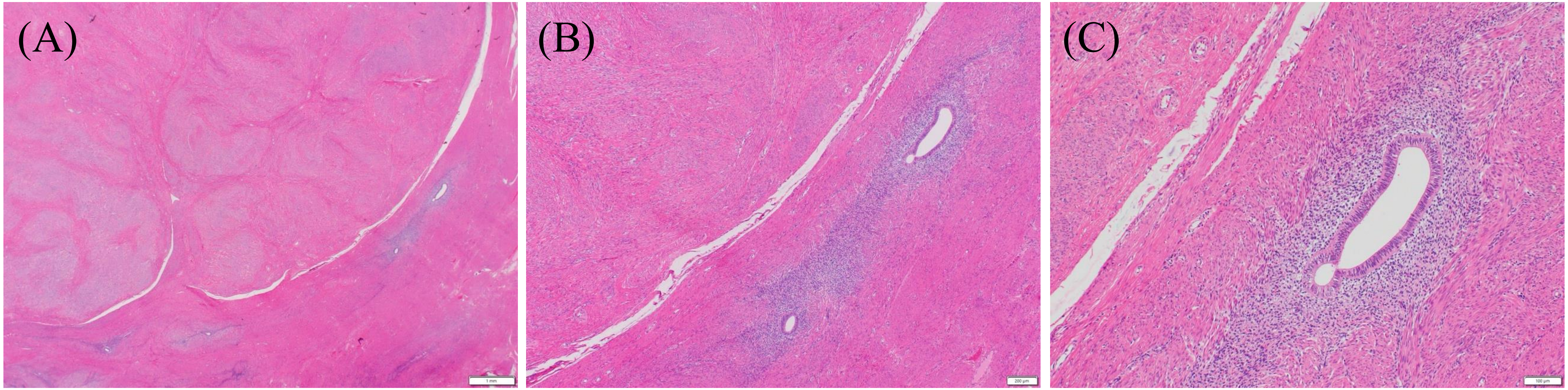

Figure S1. (A–C) Histologic findings of adenomyosis. Hematoxylin & eosin staining. Scale bars: (A) 1 mm; (B) 200 μm; (C) 100 μm.
